# The Role of the Heat Shock Protein B8 (HSPB8) in Motoneuron Diseases

**DOI:** 10.3389/fnmol.2017.00176

**Published:** 2017-06-21

**Authors:** Paola Rusmini, Riccardo Cristofani, Mariarita Galbiati, Maria E. Cicardi, Marco Meroni, Veronica Ferrari, Giulia Vezzoli, Barbara Tedesco, Elio Messi, Margherita Piccolella, Serena Carra, Valeria Crippa, Angelo Poletti

**Affiliations:** ^1^Dipartimento di Scienze Farmacologiche e Biomolecolari (DiSFeB), Centro di Eccellenza sulle Malattie Neurodegenerative, Università degli Studi di MilanoMilano, Italy; ^2^Dipartimento di Scienze Biomediche, Metaboliche e Neuroscienze, Università di Modena e Reggio EmiliaModena, Italy; ^3^C. Mondino National Neurological InstitutePavia, Italy; ^4^Centro Interuniversitario sulle Malattie Neurodegenerative, Università degli Studi di Firenze, Roma Tor VergataMilano, Italy

**Keywords:** motoneuron diseases, amyotrophic lateral sclerosis, spinal and bulbar muscular atrophy, proteasome, autophagy, chaperones, misfolded proteins, HSPB8

## Abstract

Amyotrophic lateral sclerosis (ALS) and spinal and bulbar muscular atrophy (SBMA) are two motoneuron diseases (MNDs) characterized by aberrant protein behavior in affected cells. In familial ALS (fALS) and in SBMA specific gene mutations lead to the production of neurotoxic proteins or peptides prone to misfold, which then accumulate in form of aggregates. Notably, some of these proteins accumulate into aggregates also in sporadic ALS (sALS) even if not mutated. To prevent proteotoxic stresses detrimental to cells, misfolded and/or aggregated proteins must be rapidly removed by the protein quality control (PQC) system. The small heat shock protein B8 (HSPB8) is a chaperone induced by harmful events, like proteasome inhibition. HSPB8 is expressed both in motoneuron and muscle cells, which are both targets of misfolded protein toxicity in MNDs. In ALS mice models, in presence of the mutant proteins, HSPB8 is upregulated both in spinal cord and muscle. HSPB8 interacts with the HSP70 co-chaperone BAG3 and enhances the degradation of misfolded proteins linked to sALS, or causative of fALS and of SBMA. HSPB8 acts by facilitating autophagy, thereby preventing misfolded protein accumulation in affected cells. BAG3 and BAG1 compete for HSP70-bound clients and target them for disposal to the autophagy or proteasome, respectively. Enhancing the selective targeting of misfolded proteins by HSPB8-BAG3-HSP70 to autophagy may also decrease their delivery to the proteasome by the BAG1-HSP70 complex, thereby limiting possible proteasome overwhelming. Thus, approaches aimed at potentiating HSPB8-BAG3 may contribute to the maintenance of proteostasis and may delay MNDs progression.

## Introduction

Motoneuron diseases (MNDs) are neurodegenerative diseases (NDs) in which cortical and/or spinal motoneurons are affected. They appear in sporadic or familial forms; little is known on alterations inducing sporadic MNDs, while specific gene mutations are responsible for altered RNA or protein functions in familial MNDs pathogenesis. Mutations may affect RNA/protein synthesis or activity (loss-of-function) or induce neurotoxicity (gain-of-functions). Amyotrophic lateral sclerosis (ALS) and spinal and bulbar muscular atrophy (SBMA) are MNDs mainly associated with gain-of-functions in proteins which become resistant to folding or conformationally unstable, leading to unfolding/misfolding. Misfolded proteins are prone to aggregate and neurotoxic impairing several cellular functions causing cell death. To prevent misfolded proteins toxicity, cells activate a protein quality control (PQC) system, which surveys protein folding and clears damaged substrates. The PQC system is crucial to counteract the neurotoxic events triggered by misfolded proteins and thus should be considered as a potential target for therapeutic intervention to ameliorate MNDs course.

## The Protein Quality Control System

The PQC system is composed of molecular chaperones and degradative pathways.

**Figure 1:**
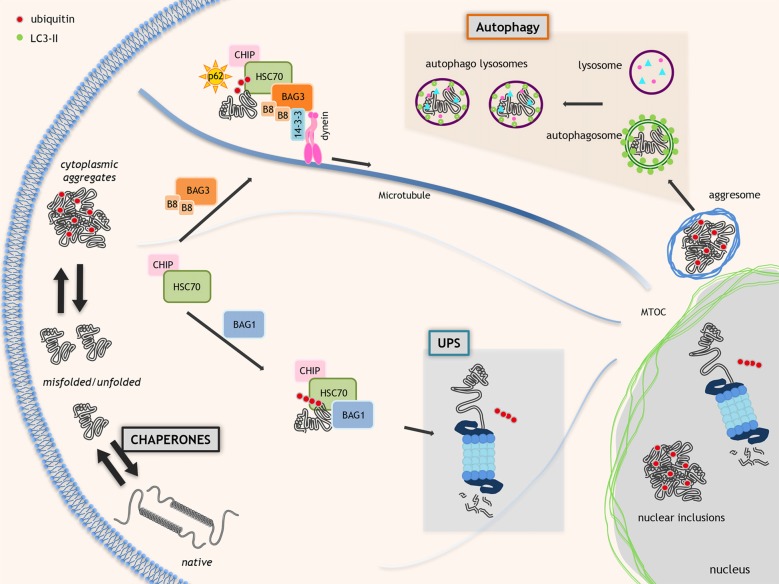
Proteostasis in neurodegenerative disorders. Molecular chaperones assist proteins to acquire the proper folding. When folding fails, the chaperones allow the ubiquitination and route misfolded proteins to degradative systems. This process is mediated by the HSC70-CHIP complex that interacts with nucleotide exchange factor (NEF)/BCL2-associated athanogenes (BAGs; HSC70 co-chaperones). BAG1 routes misfolded proteins to ubiquitin proteasome system (UPS). Alternatively, BAG3 and specific chaperone heat shock protein B8 (HSPB8; B8 in the figure) promote the degradation of HSC70 substrates via autophagy. The HSC70-CHIP interaction with BAG1 inhibits the HSP70 chaperone activity and allows misfolded proteins polyubiquitination by HSC70-binding co-factor CHIP resulting in misfolded proteins degradation via UPS. BAG3 interacts with dynein and 14-3-3 protein moving misfolded proteins to microtubule organization center (MTOC) where aggresomes are assembled. Polyubiquitinated proteins linked to HSC70-BAG3 are recognized by SQSTM1/p62 and its interaction with LC3 allows misfolded proteins insertion into autophagosomes.

Chaperones, like the heat shock proteins (HSPs), are often constitutively expressed, but also over-induced by different cell stresses (Morimoto, 2006), and include more than 150 members (grouped on the basis of their size (small HSPs, HSP40s, HSP60s, HSP70s, HSP90s and HSP100), structure and function; Kampinga and Craig, 2010). Several chaperones require co-chaperones, which act as nucleotide exchange factors (NEFs), like the BCL2-associated athanogene (BAG) family of proteins (Takayama and Reed, 2001). Several chaperones and co-chaperones are mutated in NDs or other diseases characterized by neuronal loss (Smith et al., 2015), suggesting that they are protective against neurodegeneration. Chaperones directly assist the proper folding of nascent proteins or refold denatured existing proteins. When folding fails, chaperones route unfolded, partially folded or misfolded proteins to degradation. During cell stress (i.e., presence of misfolded proteins), some HSPBs, like heat shock protein B8 (HSPB8), limit the levels of aberrant proteins escaping degradation, which can accumulate in cells (Crippa et al., 2010b; Minoia et al., 2014; Cristofani et al., 2017a; Figure 1).

The degradative pathways include the ubiquitin-proteasome system (UPS) and the autophagy (also the unfolded protein response (UPR), which relies on a specific endoplasmic reticulum associated degradation (ERAD) is part of the PQC but extensively reviewed recently (Volpi et al., 2017)). UPS and autophagy are in a finely orchestrated equilibrium, controlled by specific chaperones and co-chaperones (Gamerdinger et al., 2011a; Lilienbaum, 2013; Minoia et al., 2014; Behl, 2016; Cristofani et al., 2017a). UPS has low capacity, but high selectivity for monomeric misfolded proteins. An example of chaperone complex targeting misfolded proteins to UPS is formed by BAG1, HSP70 and the E3-ubiquitin ligase CHIP/STUB1 (Figure 1). Autophagy has high capacity, but low selectivity for substrates degrading oligo-/hetero-meric species, aggregates and damaged organelles (Klionsky et al., 2016). An example of chaperone complex targeting misfolded proteins to autophagy is the chaperone-assisted selective autophagy (CASA) complex, composed of HSPB8, BAG3, HSP70 and CHIP/STUB1. CASA complex interacts with the autophagy receptor SQSTM1/p62 which binds both ubiquitinated proteins and lipidated LC3 (LC3-II) targeting proteins to autophagosomes for degradation (Klionsky et al., 2016; Figure 1).

An imbalance of these two degradative pathways plays deleterious effects in several NDs (Kakkar et al., 2014; Ciechanover and Kwon, 2015; Nikoletopoulou et al., 2015; Senft and Ronai, 2015; Xilouri and Stefanis, 2015).

## ALS and SBMA as Models to Study Misfolded Proteins in MNDs

ALS is a MND involving brain motor cortex, brainstem and anterior horn spinal cord motoneurons. Neurons of the fronto-temporal brain regions could be involved (Robberecht and Philips, 2013), causing mixed motor and cognitive phenotype (ALS with fronto-temporal dementia or FTD). Other cells, like glial cells (astrocytes (Trotti et al., 1999; Boillée et al., 2006; Nagai et al., 2007), oligodendrocytes (Philips et al., 2013), Schwann cells (Lobsiger et al., 2009; Turner et al., 2010), microglia (Philips and Robberecht, 2011)), and muscle cells (Musarò, 2010; Onesto et al., 2011; Galbiati et al., 2014) modulate disease progression, affecting motoneuron functions and survival capability.

ALS are mainly sporadic (sALS), with only 15% familial (fALS) forms, clinically indistinguishable from sALS. fALS are linked to specific gene mutations (e.g., superoxide dismutase-1 (*SOD1*), TAR DNA-binding protein 43 (*TDP-43*), fused-in-sarcoma/translocated-in-liposarcoma (*FUS/TLS*), Sequestosome-1 (*SQSTM1/p62)*, Optineurin (*OPTN-1*), Ubiquilin (*UBQLN-2*), Valosin Containing Protein (*VCP*), TANK Binding Kinase 1 (*TBK1*) and several others (see Taylor et al., 2016 for review). Many of these gene products are autophagy-related proteins, key players of the PQC system (Ju et al., 2009; Tresse et al., 2010; Seguin et al., 2014; Taylor et al., 2016), or mislocalize and aggregate exerting proteotoxicity (Robberecht and Philips, 2013; Taylor et al., 2016). Some of these proteins are prone to aggregate in sALS even in their wild-type form (e.g., TDP-43, FUS, SQSTM1/p62, OPTN-1, UBQLN-2, etc.), suggesting that the mutation exacerbates their natural propensity to misfold (Neumann et al., 2006; Daoud et al., 2009; Bosco and Landers, 2010) and that common toxic mechanisms are involved in fALS and sALS. About 50% of fALS are linked to GGGGCC hexanucleotide (or G4C2) repeat expansions in the *C9ORF72* gene (Al-Sarraj et al., 2011; DeJesus-Hernandez et al., 2011; Renton et al., 2011); this repeat undergoes a non-canonical repeat-associated non-ATG (RAN) translation, which generates five different dipeptides (DPRs), highly aggregation-prone (Ash et al., 2013; Lashley et al., 2013; Mori et al., 2013).

SBMA differs from ALS by a slower progression rate and involves lower motoneurons, dorsal root ganglia (DRG) sensory neurons, muscle cells and different androgen-target cells in reproductive tissues. Glial cells or microglia are not involved (La Spada et al., 1991; Fischbeck, 1997; Sorarù et al., 2008; Boyer et al., 2013; Malena et al., 2013; Cortes et al., 2014; Lieberman et al., 2014). SBMA is linked to a CAG repeat expansion in the androgen receptor (*AR*) gene, which codes an elongated polyglutamine tract (polyQ) in the AR protein (ARpolyQ; La Spada et al., 1991). ARpolyQ tends to misfold acquiring neurotoxic properties (Poletti, 2004), but only after binding to its ligand testosterone (Stenoien et al., 1999; Simeoni et al., 2000; Katsuno et al., 2002, 2003). Testosterone triggers conformational changes required for AR activation possibly impaired by the polyQ.

Several studies prove that misfolded proteins accumulation in fALS, sALS or SBMA alters the degradative pathways. The UPS could be overwhelmed by an excess of misfolded proteins or clogged by the polyQ (Ciechanover and Kwon, 2015; Rusmini et al., 2016); the autophagic flux could be blocked by misfolded protein aggregates (Rusmini et al., 2013). However, the molecular steps altered by misfolded proteins in these pathways are poorly understood. Several chaperones enhance misfolded protein degradation either/both by facilitating their proteasomal degradation or/and by limiting autophagic flux alterations (Balchin et al., 2016; Rusmini et al., 2016; van Noort et al., 2017; Charmpilas et al., 2017).

## The HSPB8 Functions and Its Role in ALS and SBMA

HSPB8 is a chaperone widely distributed in several (if not all) human tissues, even if at different expression levels. In addition, HSPB8 upregulation may protect in ALS and SBMA (Carra et al., 2005, 2013; Crippa et al., 2010b; Rusmini et al., 2013). Notably, *HSPB8* mutations cause Charcot-Marie-Tooth type 2L disease, hereditary distal motor neuropathy type II (dHMN-II) or distal myopathy (Fontaine et al., 2006; Irobi et al., 2010; Ghaoui et al., 2016), diseases involving motoneurons and/or muscle cells. These mutations impair the chaperone HSPB8 activity (Kwok et al., 2011), suggesting it has a crucial role in preserving motoneuron function and viability. Here, we review experimental findings in support of this hypothesis.

**Figure 2:**
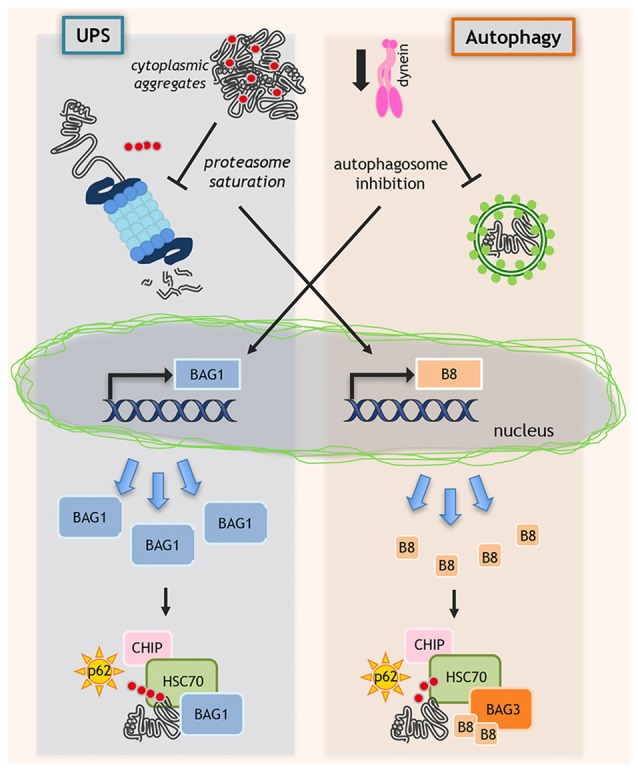
Regulation of protein quality control (PQC) system. When misfolded proteins cannot be efficiently removed by degradative pathways, misfolded proteins may accumulate and block ubiquitin proteasome system (UPS) and autophagy. In this context, proteasome saturation by misfolded proteins increases the transcription of HSPB8 (B8 in the figure) that, together with its partners BAG3 and HSC70, routes misfolded proteins to autophagy. In parallel, when dynein-mediated transport is blocked and autophagosomes formation is inhibited, still unknown factors activate the *de novo* transcription of BAG1, which binds to HSP70/CHIP and routes misfolded proteins to UPS.

Interestingly, within the spinal cord, HSPB8 is specifically found in motoneurons, and its expression declines with age (Crippa et al., 2010b) suggesting that motoneurons might become more vulnerable to misfolded protein toxicity during aging. In cultured motoneurons, HSPB8 expression is greatly induced by proteasome impairment (Figure 2; Crippa et al., 2010a,b), a condition generally occurring in MNDs. In addition, *HSPB8* mRNA expression is higher in autopsy specimens of ALS patient spinal cord than in age-matched controls (Anagnostou et al., 2010). We found a robust increase of HSPB8 protein levels in anterior horn spinal cord motoneurons surviving at end-stages of disease in transgenic (tg) ALS SOD1-G93A mice compared to wild-type mice (Crippa et al., 2010b), and this upregulation correlates with the presence of diffuse and non-aggregated mutant SOD (Crippa et al., 2010b).

HSPB8 is also highly expressed in muscle. Indeed, the genome-wide tissue analysis of RNA and protein expression (available at the Human Protein Atlas portal^1^) reports that in human skeletal muscle HSPB8 mRNA is expressed at high level, while the protein is expressed at medium levels if compared to other human tissues (Uhlén et al., 2015). In mice, HSPB8 expression in skeletal muscle dramatically increases during disease progression in ALS (Carra et al., 2013; Crippa et al., 2013a,b), and SBMA (Rusmini et al., 2015) mice. Since in these two MNDs, both motoneurons and myoblasts are target of misfolded protein toxicity, the increased HSPB8 expression may contribute to enhance the aberrant proteins clearance from muscle to improve cell survival. This hypothesis is supported by a recent report showing that the protein ICP10PK, the herpes simplex virus type 2 (HSV-2) homolog of HSPB8, delays disease onset and slows down progression rate of tg ALS SOD1-G93A mice (Aurelian et al., 2012). These affects are associated to reduced damages at neuromuscular junctions, and to increased motoneuronal survival (Aurelian et al., 2012). We demonstrated that the overexpression of *HSP67Bc*, the fly functional ortholog of *HSPB8*, exerts protective effects in two *Drosophila melanogaster* models of ALS. HSP67Bc overexpression prevented the mislocalization of a neurotoxic mutant TDP-43 protein (Ritson et al., 2010), while HSP67Bc downregulation correlated with increased TDP-43 and polyubiquitinated proteins accumulation, worsening the eye phenotype, of mutant TDP-43 flies (Crippa et al., 2016). HSP67Bc also rescued from pupae lethality flies overexpressing the ALS-associated 35 kDa TDP-43 fragment (TDP-35; Crippa et al., 2016). Thus, HSPB8 upregulation protects against misfolded protein-mediated toxicity in ALS models.

At cellular levels, the HSPB8 protective effects associate to its capability to facilitate misfolded proteins autophagic degradation. Indeed, HSPB8 could remove the blockage of autophagic flux found in several NDs (Rusmini et al., 2013, 2016; Giorgetti et al., 2015; Crippa et al., 2016). The HSPB8 pro-degradative activity was demonstrated with several different neuropathogenic proteins, like polyQ proteins (ARpolyQ, huntingtin-polyQ, ataxin-3-polyQ), beta-amyloid, alpha-synuclein, ALS proteins mutant SOD1 and TDP-43 fragments (Chávez Zobel et al., 2003; Wilhelmus et al., 2006; Carra et al., 2008a,b; Crippa et al., 2010b, 2016; Bruinsma et al., 2011; Seidel et al., 2012; Rusmini et al., 2013), and on five different RAN translated DPRs from the *C9Orf72* gene linked to ALS and FTD (Cristofani et al., 2017b). In most cases, HSPB8 down-regulation resulted in increased accumulation of these mutant proteins and DPRs, supporting its role in the misfoded protein clearance (Crippa et al., 2010b, 2016; Rusmini et al., 2013; Cristofani et al., 2017b).

Moreover, increasing genetic and experimental evidences suggest that the RNA-protein inclusions accumulating in ALS and similar NDs may arise from the conversion of dynamic ribonucleoprotein complexes, stress granules (SGs), into amyloid-like aggregates. In particular, misfolded proteins accumulating in SGs promote their conversion into aggregates. Intriguingly, the PQC system response named “granulostasis”, surveys SG composition and maintains their dynamic behavior. One of the key players of granulostasis is the HSPB8-BAG3-HSP70 complex (Ganassi et al., 2016; Carra et al., 2017; Mateju et al., 2017).

## The Molecular Mediators of The HSPB8 Pro-Autophagic Activity in Motoneurons

In line with the proposed pro-autophagic activity of HSPB8, autophagy, but not proteasome inhibition blocks, the HSPB8 pro-degradative activity (Crippa et al., 2010b, 2016; Rusmini et al., 2013). Mechanistically, HSPB8 facilitates the autophagic clearance of misfolded protein by associating with BAG3 (in a 2:1 ratio) and HSP70 (Carra et al., 2008b). The HSPB8-BAG3-HSP70 complex allows the cargoes delivery (i.e. misfolded proteins) to autophagy for degradation (Figure 1). BAG3 is essential in the complex, and its loss leads to a fast HSPB8 degradation (Carra et al., 2008b). In physiological conditions, the HSPB8-BAG3-HSP70 complex is essential at muscle level for Z-disk maintenance, where it is induced in response of acute physical exercise and of repeated mechanical stimulation (Ulbricht et al., 2015). These conditions generate a large excess of damaged proteins (e.g., actin) by post-translational modifications (carbonylation, nitrosylation), and the HSPB8-BAG3-HSP70 complex recognizes these damaged proteins and, by interacting via HSP70, with the E3-ubiquitin ligase CHIP/STUB1, forms the CASA complex. Here, CHIP/STUB1 ubiquitinates the target substrate allowing its SQSTM1/p62 recognition and insertion into the autophagosomes for degradation (Arndt et al., 2010). In skeletal muscle, the CASA complex directly interacts with DNAJB6 (of the DNAJ/Hsp40 family; Sarparanta et al., 2012), an HSP70 co-chaperone that suppresses aggregation of several misfolded proteins involved in NDs (Hageman et al., 2010). Indeed, *DNAJB6* mutations cause Limb-girdle muscular dystrophies (LGMDs) in which a less-effective anti-aggregation activity of DNAJB6 has been found. In LGMDs patient muscle biopsies, DNAJB6 and CASA complex proteins aggregation and accumulation are observed, suggesting that the pathogenesis is also mediated by CASA dysfunctions (Sandell et al., 2016).

We demonstrated an important role of the CASA complex in motoneurons under pathological conditions, since in ALS cell models, this complex clears misfolded mutant SOD1 accumulating into motoneurons (Crippa et al., 2010a,b). While HSPB8 appears to be the limiting factor of this complex, BAG3 mediates its formation acting as a scaffold which interacts by its N-terminus with HSPB8 and by its C-terminus with HSP70 (which binds CHIP/STUB1). BAG3 contains PXXP motif, adjacent to the BAG domain, which binds dynein (Merabova et al., 2015), and two binding sites for the 14-3-3 protein, which stabilizes the BAG3 and dynein interaction (Mccollum et al., 2010; Behl, 2011, 2016; Gamerdinger et al., 2011a,b; Xu et al., 2013; Jia et al., 2014; Merabova et al., 2015). Dynein allows the efficient transport of the entire BAG3-multi-heteromeric complex at the site of autophagosomes assembly (Arndt et al., 2010; Crippa et al., 2010b; Merabova et al., 2015; Figure 1).

Interestingly, when dynein mediated transport is genetically (siRNAs) or pharmacologically (EHNA) blocked, the HSP70-CHIP cannot bind HSPB8-BAG3 complex to dispose misfolded proteins (ARpolyQ, mutant SOD1 and truncated TDP-43) via autophagy (Cristofani et al., 2017a). Here, still unknown factors activate the *de novo* transcription of another NEF/BAG, the BAG1, which binds HSP70/CHIP re-routing misfolded proteins to UPS (Behl, 2011, 2016; Gamerdinger et al., 2011a; Cristofani et al., 2017a). Indeed, BAG1 exogenous overexpression facilitates proteasomal removal of ARpolyQ. When dynein transport is inhibited, the pro-degradative BAG1 activity is blocked by proteasome inhibitors, but not by autophagy blockers (Cristofani et al., 2017a; Figure 2).

The BAG3/BAG1 ratio is the key factor that determines the fate of misfolded protein degradation via UPS or autophagy. HSPB8, being a chaperone “holder”, would bind to client proteins keeping them in a competent state for further processing by the BAG3-HSP70 complex. Increases in HSPB8 levels restore a deficient autophagic flux and ensure proper targeting of misfolded proteins to autophagy for clearance (Figure 2).

## HSPB8-BAG3 Induction as Possible Therapeutic Approach for MNDs

Since HSPB8 overexpression is sufficient to restore autophagy, HSPB8 acts as a limiting factor for misfolded proteins autophagic degradation. Thus, small molecules acting as HSPB8 inducers may be of therapeutic interest in MNDs (Figure 2). Indeed, estrogens are physiological HSPB8 inducers, and selective estrogen receptor modulators (SERM) may differentially control its expression (Sun et al., 2007; Piccolella et al., 2017). To find FDA-approved drugs and natural compounds inducers of HSPB8 expression, we performed a high throughput screening (HTS) based on the human HSPB8 promoter controlling luciferase expression. We identified colchicine and doxorubicin as potent HSPB8 inducers and autophagy facilitators of removal of insoluble TDP-43 species (Crippa et al., 2016). Colchicine and derivatives might represent useful compounds to be tested in ALS models. Besides colchicine, which may have side effects, we found another HSPB8 inducer, the autophagic stimulator trehalose (Rusmini et al., 2013), already tested with positive results in several mice models of NDs (Tanaka et al., 2004; Davies et al., 2006; Rodríguez-Navarro et al., 2010; Perucho et al., 2012; Schaeffer and Goedert, 2012; Castillo et al., 2013; Du et al., 2013; Sarkar et al., 2014; Zhang et al., 2014; He et al., 2016).

Recently, it was shown that trehalose was also able to upregulate BAG3 expression (Lei et al., 2015). Whether trehalose protective effect is partly mediated by HSPB8 and BAG3 induction, in mice has still to be tested and represents an attractive hypothesis for future studies. Other compounds able to induce BAG3 expression has not been tested in MNDs models. Several evidences demonstrate that besides its role in autophagy, both HSPB8 and BAG3 modulate intracellular pathways involved in apoptosis or development altered in several tumors, and pharmacological BAG3 upregulation is obtained with proteasome inhibitors, TNF-related apoptosis-inducing ligand, fludarabine, cytosine arabinoside and etoposide, compounds used in chemotherapy, indicating BAG3 as a mediator of therapy resistance (Romano et al., 2003; Chiappetta et al., 2007; Rapino et al., 2014).

Thus, from preclinical studies, the pharmacological induction of key molecules like HSPB8 or BAG3 represents a novel and attractive target to treat MNDs. However, given the ability of these proteins to modulate major biological processes, the side-effects of their induction on other intracellular pathways remain to be elucidated.

## Conclusions

Data collected in recent years clearly demonstrated that an increased PQC system activity protects against proteotoxic stresses induced by MNDs associated with the accumulation of misfolded proteins. Amongst the several PQC system components, the chaperone HSPB8 has attracted great attention, since it exerts a potent pro-degradative activity on misfolded proteins facilitating their removal via autophagy and preventing their intracellular accumulation. HSPB8 acts in a molecular complex involved in the fine tuning of the equilibrium between the UPS and autophagy. By this mechanism, HSPB8 also prevents the delivery of excessive amounts of misfolded proteins to UPS by routing them to autophagy. Based on these findings, the pharmacological induction of HSPB8 in MND affected cells should represent a promising therapeutic approach to counteract disease onset and progression.

## Author Contributions

PR, RC, MG, SC, VC and AP designed and wrote the manuscript and critically discussed all sections of the minireview article. In addition RC, prepared the figures. MEC, MM, VF, GV, BT, EM, and MP critically revised the important intellectual content of the manuscript and the figures. All authors have provided final approval of the version to be published.

## Conflict of Interest Statement

The authors declare that the research was conducted in the absence of any commercial or financial relationships that could be construed as a potential conflict of interest.

## Footnotes

^1^www.proteinatlas.org
